# Will Nicotine Genetics and a Nicotine Vaccine Prevent Cigarette Smoking and Smoking-Related Diseases?

**DOI:** 10.1371/journal.pmed.0020266

**Published:** 2005-09-27

**Authors:** Wayne D Hall

## Abstract

Hall argues that the preventive use of genetic and vaccine biotechnologies is a superficially attractive tobacco policy option of doubtful efficacy, cost-effectiveness, and ethicality.

Biotechnologies that aim to prevent smoking and tobacco-related disease may emerge as unintended by-products of research on the genetics of nicotine dependence and the effectiveness of nicotine vaccines for smoking cessation. For many advocates of tobacco control, any discussion of the genetics of smoking is anathema because of the tobacco industry's use of Fisher's [1] genetic hypothesis to argue that cigarette smoking did not cause lung cancer [2,3]. Advocates of tobacco control say that the goal of tobacco policy should be to eliminate cigarette smoking by imposing high taxes on tobacco products, preventing the tobacco industry from promoting its products, restricting access to tobacco and opportunities to smoke, and minimising nonsmokers' exposure to environmental tobacco smoke [4]. These policies have substantially reduced smoking prevalence in the developed countries that have adopted them [4].

Even if one agrees with these policies, as I do, it would be unwise to ignore evidence that genetic factors play a role in smoking initiation and persistence or neurobiological explanations of why some smokers find it so difficult to quit. Indeed, the past misuse of genetic research on nicotine and the possible public misunderstanding of the role of nicotine vaccines make it all the more important for public health practitioners and genetic and neurobiological researchers to be well acquainted with some of the superficially attractive but mistaken ways in which this work may be used.

In this short paper, I briefly explain why it would be unwise to use genetic and neurobiological knowledge to prevent cigarette smoking and tobacco-related disease. However implausible these uses may seem to those who are well informed about the genetics of tobacco use or tobacco-control policy, it is the preventive uses of genetic information and nicotine vaccines that most excite the interest of the media and the public. The major challenges that these approaches face need to be widely understood if we are to prevent these superficially attractive but controversial uses from undermining effective control policies and the development of better methods of helping smokers to quit.

## The Genetics of Smoking

Twin studies of cigarette smoking [5,6] estimate that the heritability of smoking initiation is 50% and that for smoking persistence is 70% [5,7,8]. There are a number of plausible “candidate genes” that predict an increased risk of nicotine dependence [5,8]. These include polymorphisms that affect nicotine metabolism, as well as dopamine receptors and transporters that mediate reward in the nucleus accumbens [9]. The most plausible hypothesis is that multiple genes are involved in smoking initiation and persistence [5,7,10,11], each of which only modestly increases the risk of developing dependence. The relative risks for the alleles that have been most consistently associated with smoking initiation, adoption, persistence, and cessation are typically less than 1.30 [5], well within the range of associations observed between polymorphisms and other human diseases, namely, 1.2–1.5 [12].

## Predictive Testing for Genetic Risks of Nicotine Dependence

The predictive testing of genetic risk dependence is one of the first uses that journalists often suggest for research on the genetics of nicotine dependence. In this scenario, the population would be screened for susceptibility alleles with the aim of giving preventive behavioural and pharmacological interventions to individuals who are at higher genetic risk of smoking [13]. There is an obvious objection to this proposal: that it is not good public health policy to encourage people to smoke tobacco, regardless of their genetic risk of dependence [7].[Fig pmed-0020266-g002]

**Figure 1 pmed-0020266-g002:**
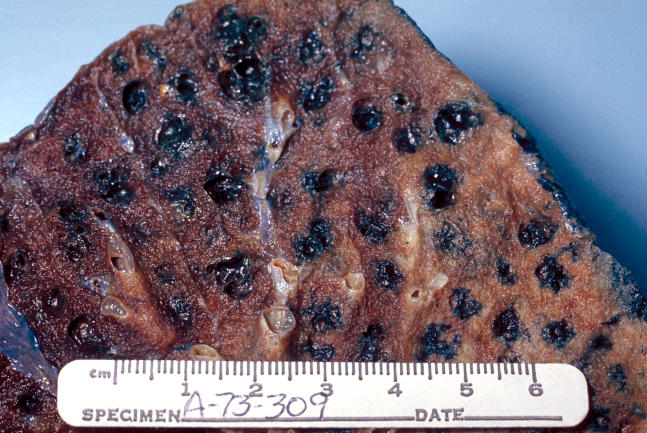
Gross Pathology of the Lung Showing Centrilobular Emphysema Characteristic of Smoking (Photo: Edwin P. Ewing, Jr./Centers for Disease Control and Prevention)

An alternative rationale is that screening would allow individuals who were at highest genetic risk of nicotine dependence to make an informed decision to avoid tobacco smoking. Even if we value individual autonomy highly, genetic screening for nicotine dependence is unlikely to be a good policy for the following reasons [14].

First, when multiple genes predispose to a condition, individual susceptibility alleles only predict a very modestly increased risk of dependence [15]. Testing multiple alleles would improve prediction if the results of the multiple tests were combined [16,17], but the larger the number of genes that are involved in disease susceptibility, the less useful most individuals will find information about their genotype. This is because as the number of alleles increases, the risk distribution tends to the log normal [17], a distribution in which the number of persons with very high risk combinations of multiple genes is very small and in which the majority will be at average genetic risk [7,16].

Second, the above means that a very large number of individuals would need to be screened to identify the few at highest risk [18,19]. For example, Yang et al. [20] simulated screening for 5 susceptibility alleles, each with a relative risk (RR) ranging from 1.5 to 3.5 and a prevalence in the population between 0.10 and 0.25 (all within the range of empirical data). One of these alleles was assumed to interact with an environmental exposure with a prevalence of 15% and a RR of 2.0. Their simulation showed that those who screened positively on all five genes had an 81% chance of developing the disease; this rose to 89% if they had also been exposed to the environmental risk factor. However, these risks applied to only four persons in 100,000, and only 79 in 100,000 would have a greater than 50% risk.

Third, predictive genetic testing may have unintended adverse effects. This would be the case, for example, if testing adolescents for susceptibility to nicotine dependence increased their preparedness to initiate smoking—as could happen, for example, if they were prompted to test the accuracy of the genetic predictions by smoking [7]. [Fig pmed-0020266-g001]

**Figure pmed-0020266-g001:**
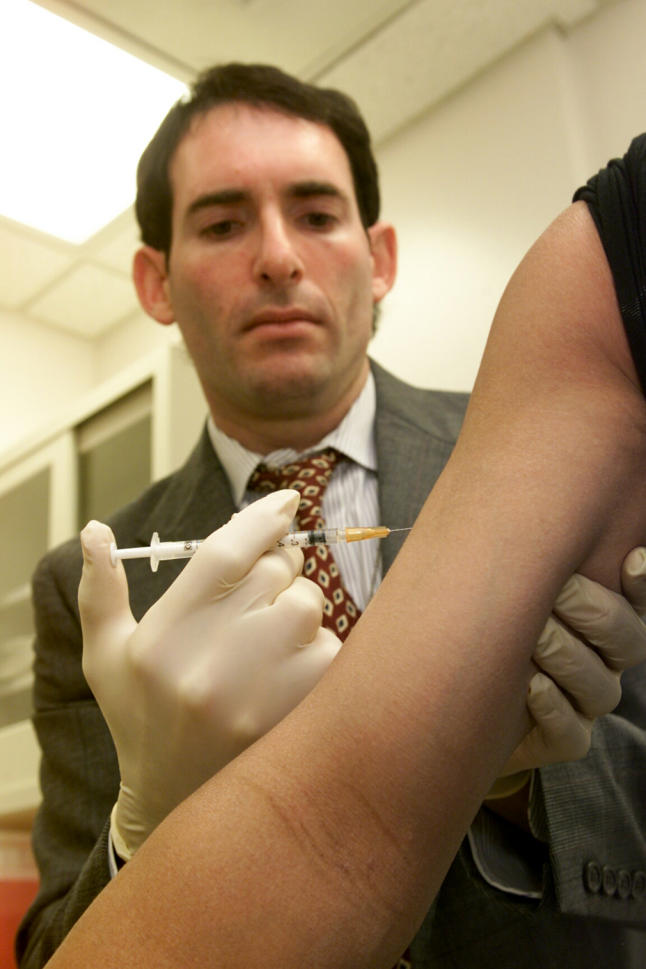
Vaccination against nicotine could reduce relapse to smoking in abstinent smokers (Photo: Bill Branson/National Cancer Institute)

Fourth, screening is only ethically justifiable if there is an effective intervention to prevent the disorder in those who are identified as being at increased genetic risk [15,21]. “Avoid smoking” is good health advice, regardless of one's genetic susceptibility. The prospect of combining genetic screening with some preventive intervention sounds a more appealing option, especially when such an intervention—a vaccine against nicotine—is being developed and trialled by three pharmaceutical companies for smoking cessation [22].

## Preventive Use of a Nicotine Vaccine

A “nicotine vaccine” induces the immune system to produce antibodies that bind to nicotine and prevent it from crossing the blood–brain barrier to act on receptors in the brain [23–25]. Animal studies have shown that attaching nicotine to a suitable antigenic protein (e.g., [23,24]) produces antibodies that have a high affinity for nicotine [23,25]. Vaccination of animals attenuates nicotine effects [24], abolishes nicotine self-administration [23], and suppresses dopamine release in the nucleus accumbens [26].

Active vaccination against nicotine could reduce relapse to smoking in abstinent smokers by attenuating the pharmacological effects of nicotine during the first few months after quitting, when most smokers relapse [25]. A nicotine vaccine could be circumvented by increasing the dose of nicotine, but attenuating the rewarding effects of nicotine may nonetheless be enough to reduce relapse rates in smokers by making a lapse less likely to lead to a return to daily smoking [22,25]. This promising immunological technology for improving the success of smoking cessation is currently in trials [22,25].

The term “vaccine” inevitably prompts journalists to ask about its possible preventive use. Misconceptions that a vaccine will produce lifelong immunity against nicotine may prompt parents to vaccinate their children [27]. As minors, children would not be legally able to consent to vaccination, but since parents already make choices for their children about other vaccines and interventions that affect their lives (e.g., their diet and education), some have argued that vaccination against nicotine and other drugs is simply another decision that parents should be able to make on behalf of their children [27]. This argument is likely to be contested by civil libertarians and by adolescents who disagree with their parents' wishes. One can expect the tobacco industry to amplify such dissenting views.

Even if we set aside the ethical issues, there are major practical obstacles to the preventive use of a nicotine vaccine in children. First, the limited period of protection provided by existing vaccines would require booster injections, perhaps every two or three months throughout adolescence [28]. Second, the fact that the vaccine could be circumvented by using higher doses of nicotine means that vaccination could be counterproductive if adolescents were prompted to test its efficacy. Third, it would be costly to universally vaccinate children against nicotine with a vaccine that would probably have only modest preventive efficacy. These obstacles (and the high regulatory hurdles that such a vaccine might be expected to have to leap when used preventively in children) make it unlikely that universal nicotine vaccination would be publicly funded.

“Indicated vaccination” of “high risk” adolescents seems a more plausible option because it would be much less expensive to only vaccinate young people who are at “high genetic risk” of smoking. The feasibility of this approach is also doubtful, given the likely low predictive validity of genetic screening for smoking risk (outlined above), the doubtful preventive efficacy of a nicotine vaccine, and the possible adverse effects of vaccination, such as stigmatisation of those who screen positive, and discrimination against them by third parties, such as life or health insurance companies.

The “off-label” use of a nicotine vaccine by a physician acting at the request of a parent is the most likely way in which a vaccine will be used preventively. It is difficult to see how this can be prevented if a nicotine vaccine is approved for therapeutic use, other than by education of physicians and parents about the limitations of this approach.

## Screening for Genetic Susceptibility to Tobacco-Related Diseases

Genetic factors also appear to play a role in susceptibility to many nonfamilial types of cancer, although there is disagreement about how large a role this is [29–32]. There is evidence that polymorphisms that affect the metabolism of carcinogens in tobacco smoke and repair of damage to DNA may increase the risk of smokers developing lung cancer [33–35]. There are also indications that polymorphisms may affect the likelihood of smokers developing heart disease [36] and chronic obstructive lung disease (Figure 1) [37].

Many ambivalent smokers may be attracted by the superficially plausible idea that they could continue to smoke with impunity if they did not have any of the alleles that predict an increased risk of smoking-related diseases [38]. However, this type of screening is very unlikely to work, for reasons that need to be widely understood by the community.

There are major practical obstacles to the preventive use of a nicotine vaccine in children.

First, if, as seems most likely, multiple genes are involved in susceptibility to multiple tobacco-related diseases, then the ability to predict tobacco-related disease risk from genetic tests may not improve on the prediction of disease risk from smoking status.

Second, cigarette smoking causes multiple diseases, with lung and other cancers, heart disease and chronic obstructive lung disease being the most prevalent. This means that predicting one's genetic risk of only the most common tobacco-related diseases would involve testing a large number of polymorphisms.

Third, because multiple susceptibility alleles would have to be tested for multiple diseases, most smokers would be at increased genetic risk of developing at least one smoking-related disease [38]. For example, if we screened for six different susceptibility alleles (each with a RR of 1.5, a prevalence of 10%, and multiplicative risks) for each of five major tobacco-related diseases (lung cancer, coronary heart disease, chronic lung disease, other cancers, and stroke), then only 3% of the screened population would not be at increased risk of developing any of the diseases. Conversely, 97% of smokers would be at increased risk of developing at least one of these diseases if they continued to smoke.

## Conclusion

The preventive use of genetic and vaccine biotechnologies—screening the population for genetic susceptibility to nicotine dependence, vaccinating children who do not smoke against the effects of nicotine, and screening smokers for polymorphisms that predict increased susceptibility to tobacco-related diseases—are superficially attractive tobacco policy options that are of doubtful efficacy, cost-effectiveness, and ethicality. We must ensure that the speculative use of these technologies does not undermine effective tobacco-control policies and the development of more effective ways of helping cigarette smokers to quit.

## Abbreviation

RR: relative risk
